# Case Report: Bronchial artery embolization and chemoradiotherapy for central squamous cell lung carcinoma with rapid regression

**DOI:** 10.3389/fonc.2022.1026087

**Published:** 2022-12-14

**Authors:** Siqi Zhou, Jianxin Zhang, Xue Meng, Yingtao Meng, Xiao Han

**Affiliations:** ^1^ Department of Oncology, Shandong First Medical University and Shandong Academy of Medical Sciences, Jinan, Shandong, China; ^2^ Department of Radiation Oncology, Shandong Cancer Hospital and Institute, Shandong First Medical University and Shandong Academy of Medical Sciences, Jinan, Shandong, China

**Keywords:** lung cancer, hemoptysis, interventional embolization, combined treatment, case report

## Abstract

**Background:**

Interventional embolization is a common treatment for hemoptysis, one of the complications of lung cancer. However, there are no official guidelines for the use of this method in antitumor therapy.

**Case Description:**

Herein, we describe a case of a patient who was pathologically diagnosed as central squamous cell lung cancer. The patient received chemotherapy, interventional embolization and radiotherapy successively. The tumor regressed rapidly within 48 hours of receipt of interventional embolization. Furthermore, the tumor decreased by more than 50% in size within 7 days during radiotherapy. Unfortunately, the patient has since developed lymph node metastases and remains under treatment.

**Conclusions:**

Thus, finding the suitable blood vessel embolized may be a suitable option to reduce the local tumor load and can be considered as antitumor therapy in combination with other treatments. The patient’s theoretical hypoxia state after interventional therapy still produced a good tumor regression after radiotherapy. However, so far, no related studies have reported the changes of tumor immune microenvironment in human body after intervention and radiotherapy.

## Introduction

Interventional embolization is a minimally invasive treatment that aims at blocking blood flow by placing embolic materials to achieve the purpose of treatment. It is often used to treat patients with hemoptysis (1, 2) but is rarely used as an antitumor therapy. However, based on its mechanism of action, bronchial arterial embolization (BAE) as an auxiliary means of local antitumor therapy may be capable of providing a good response (3–8). Here, we report a case of tumor remission achieved through this intervention when used in combination with other treatments. Although it is not a conventional clinical treatment, considerable therapeutic effect was seen in this case. We present the following case in accordance with the CARE reporting checklist.

## Case presentation

**Figure 1:**
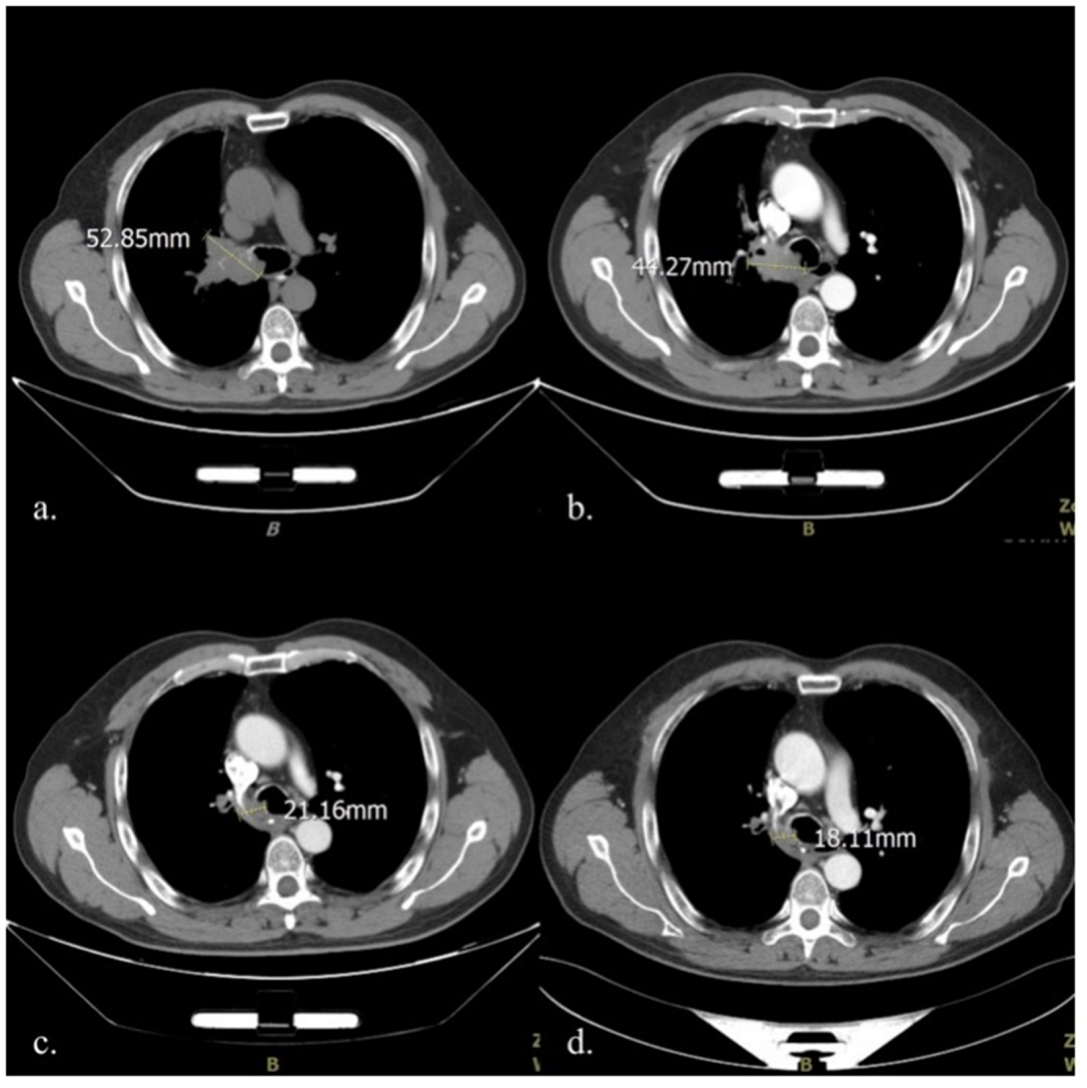
Comparison of mediastinal window CT images in four different periods: **(A)** Before treatment initiation (2020-10-12); **(B)**. After two cycles of chemotherapy (2020-11-25); **(C)** At the end of the 5^th^ fractionation of radiotherapy (2020-12-9); **(D)** At the end of the 10^th^ fractionation of radiotherapy (2020-12-16). CT, computed tomography.

A 57-year-old Chinese man was admitted to the respiratory department at a regional hospital owing to hemoptysis and cough, no obvious cause was identified, and the patient denied having chest pain, fever, shiver, and other concomitant symptoms. The patient’s social history was as follows: 60-pack years and 30 years of significant alcohol intake; however, he had been sober for 10 years. There was no other relevant medical history of note, and the patient revealed no genetic, congenital, or developmental abnormalities. Unfortunately, the family genetic history was not provided. The patient had not received any prior treatment for these symptoms. Physical examination revealed that the superficial lymph nodes of the whole body were not palpable or enlarged. The breathing sounds of both lungs were clear, and no coarse or fine crackles were heard. Computed tomography (CT) scan revealed an irregular large soft tissue mass in the right hilar region, invading the right main bronchus, narrowing, and truncating with atelectasis. The tumor diameter was 52.85 mm (mediastinal window images were uniformly used for measurement and comparison) with atelectasis (**Figure 1A**). Histological examination of a transbronchial specimen confirmed that the tumor was a squamous cell carcinoma (SCC). No lymph node metastases or distant organ metastases were identified. Based on the 8^th^ Edition Lung Cancer Stage Classification, it was diagnosed as right central lung SCC with right upper lobe atelectasis (stage cT3N0M0, IIB) (9).

As the tumor was close to the carina, a multidisciplinary team (MDT) consultation was undertaken; the team consisted of a medical oncologist, a thoracic surgeon, a radiologist, and a pathologist. As the MDT concluded that the risks associated with the surgical resection of the tumor were very high, it was not considered at the time. A treatment plan was developed for induction chemotherapy with sequential thoracic radiotherapy since the large diameter of the tumor and vascular invasion. Paclitaxel (albumin-bound) 400 mg D1 combined with carboplatin 500 mg D1 on a 21-day cycle was used for induction chemotherapy. The first treatment cycle began on October 16, 2020, and the two-cycle efficacy was evaluated as stable disease **(** **Figure 1B** **)**.

At the end of the first round of chemotherapy, the patient again presented with cough with hemoptysis in the early morning of the 3^rd^ day; the blood was dark red and of approximately 10-15 ml. Routine blood test revealed that the bleeding was not caused by thrombocytopenia, a common adverse reaction to chemotherapy. Bronchial artery embolization was performed twice during chemotherapy to address the symptoms. The first embolization was performed on October 19, 2020. The interventional doctor injected the contrast medium into the thickened artery— the trunk of the right middle and lower lobe bronchial artery; this helped to clearly stain the tumor, and the staining disappeared after poly vinyl alcohol (PVA) microspheres measuring 300–500 mm in diameter was injected into the arteries. However, the results were unsatisfactory, and the patient’s hemoptysis did not reduce in intensity following the procedure. A second embolization was performed 6 weeks later (December 2, 2020). The PVA microspheres with a diameter of 100-300mm and two coils with diameter 2cm/crimp diameter 2cm (2-2) were injected into the right upper lobe bronchial artery; and four coils with diameter 2cm/crimp diameter 2cm (2-2) were injected into the right middle lobe bronchial artery. The patient’s hemoptysis disappeared; no chemotherapeutic drugs were used in either procedure. The patient tolerated the intervention, and there were no adverse reactions, such as fever, headache, nausea, or vomiting. On the second day after embolization, intensity-modulated radiation therapy (IMRT) was initiated at a dose of 60 Gy for 30 fractions. On December 9, 2020, enhanced CT performed during radiotherapy to reposition the patient revealed that the tumor had significantly reduced in size to 21.16 mm **(** **Figure 1C** **)**. On December 16, 2020, after the 10^th^ radiotherapy fractionation, an enhanced CT scan revealed that the tumor had reduced to 18.11 mm in size. It remained this size for 4 months **(** **Figure 1D** **)**. Unfortunately, the patient has since developed lymph node metastases (occurred 3 months after radiotherapy) and remains under treatment. The patient provided informed consent for publication of this case report.

## Discussion

**Figure 2:**
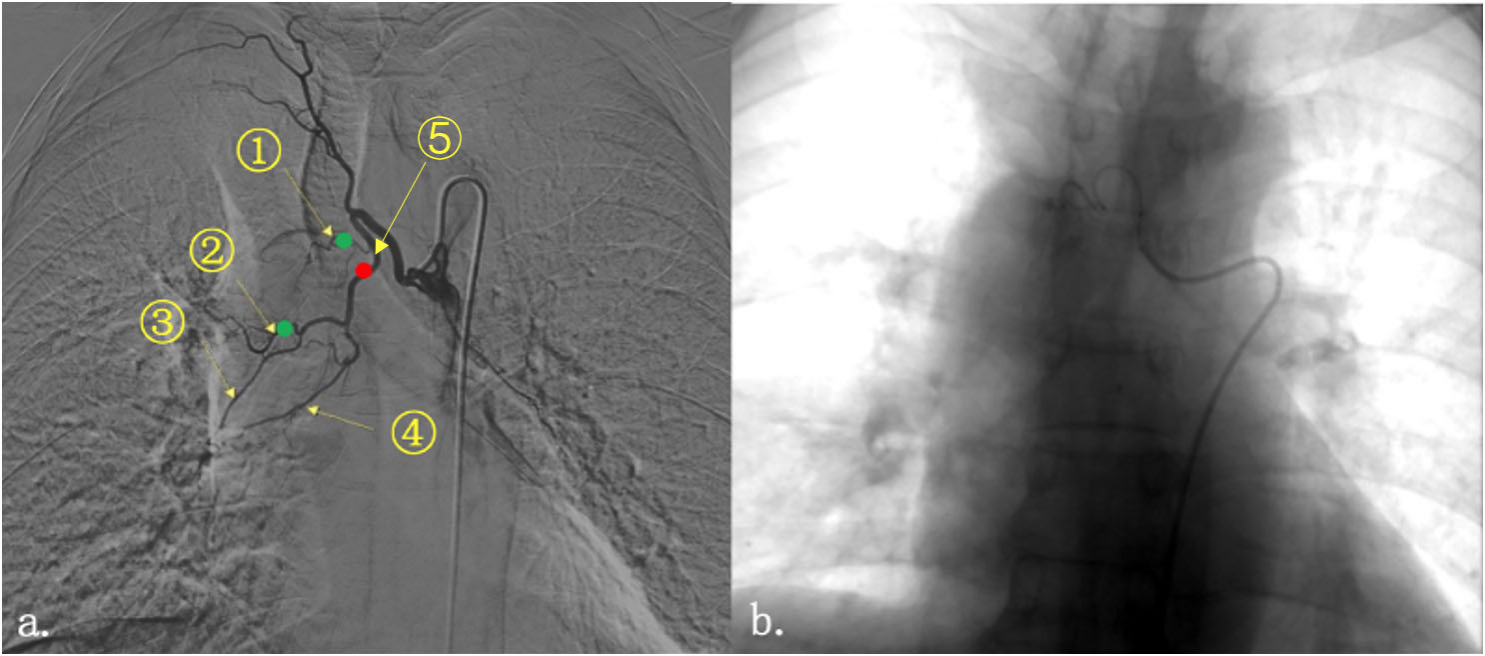
Imaging of tumor under contrast medium perfusion. Right bronchial artery angiography showed that the tumor at the right hilum was mainly supplied by two blood vessels **(A)**. They are (1) the right upper lobe bronchial artery; (2) the right middle lobe bronchial artery; (3) the right bronchial artery flowing through part of the middle lobe and lower lobe; (4) the right inferior lobe bronchial artery; and (5) the trunk of the right middle and lower lobe bronchial artery. The red dot represented the general location of the first embolization; and the green dot represented the location of the second one. **(B)** The bronchial artery was embolized by injection of microspheres and coils until the vascular enhancement image disappeared.

In this case, the SCC of the lung markedly reduced after interventional therapy. Albumin-bound paclitaxel-carboplatin combination is a standard chemotherapy regimen for SCC of the lung. The patient first received two cycles of this regimen as induction chemotherapy, with resultant stabilization of disease (10). The hemoptysis symptoms worsened during chemotherapy, indicating that the tumor was not satisfactorily controlled; two BAEs were therefore performed. Different blood vessels were used in both embolization processes. The first embolization was performed on the right lower lobe bronchial artery. Although the interventional doctor identified the responsible vessels on injection of contrast media before and after embolization, the hemoptysis was not completely relieved. Angiography of the tumor and its vasculature was repeated; it was noted that the tumor was located at the junction of the upper and middle lobes of the right lung **(** **Figure 2** **)**. Therefore, the small right upper lobe bronchial artery was embolized the second time, and the embolization of the middle lobe bronchial artery was improved. On the second day after the surgery, the patient expectorated soft, grayish-red tissue (December 4, 2020). This was pathologically confirmed as SCC, and it was reported as being necrotic and friable. This suggests that the rapid reduction of the tumor likely occurred within 48 hours after the interventions discussed in this case study. Successive imaging during radiotherapy also confirmed this.

The maximum diameter of the tumor decreased by more than half (from 44.27 mm to 21.16 mm) on day 7 after therapeutic intervention following the administration of only 5 fractions of radiation (cumulative dose: 10 Gy), considerably less than the current, effective antitumor dose recommended (11). Therefore, we hypothesize that the short-term effect on the tumor is related to bronchial artery embolization. Vascular occlusions are caused by placing embolic material upstream of the target blood vessels to occlude it and prevent hemoptysis. In fact, interrupting the blood supply can block the supply of nutrients necessary for tumor growth and enable necrosis. However, it is difficult to block the blood supply entirely in the clinical setting. Thus, embolization is typically used as a treatment for hemoptysis, and it is rarely used in local antitumor therapy.

Tumors can develop new vascular branches when the primary blood supply is blocked. These new branches are often very small and difficult to observe with the current imaging techniques. Even if a blood vessel is identified, its small lumen diameter makes the surgical procedure challenging. In a study by Fujita et al. (7), bleeding did not stop immediately in 18% patients with hemoptysis who received BAE. This was due to the incomplete embolization of the contributory vessels. Some large tumors invade the mediastinum and the thoracic wall, precluding complete embolization of the contributory arteries (7).

Furthermore, it is challenging to choose the appropriate embolus. Arteries are elastic, and if the embolization procedure is not secure, blood may flow through, resulting in embolization failure. Many types of embolic agents are clinically used, each with distinct properties. Because of its small diameter, ethiodized oil injection can cause complications, such as spinal cord injury and cerebral embolism. There is also the disadvantage of incomplete embolization. Gelatin sponge particles have good flexibility, excellent transport ability, and better fusion with the target vessels; thus, embolization is thorough. However, due to their biodegradability, blood flow is easily restored (12, 13), necessitating multiple interventional therapies to be performed (14, 15).

In China, some physicians perform palliative treatment through interventional therapy combined with chemoembolization by administering chemotherapeutic drugs directly into the tumor-feeding artery. This can significantly increase the local drug concentration in the tumor and reduce systemic adverse reactions (16). Zhao RF et al. (17) studied 50 patients with lung carcinoma who were administered chemotherapeutic drugs with Embosphere^®^ Microspheres (Merit Medical, Utah, USA) into target vessels. The efficacy of the intervention was evaluated one month after the third treatment. An overall effective rate of 88% was observed—9 patients (18%) had complete responses, 35 (70%) had partial responses, and 6 (12%) had no change. A prospective study also achieved good responses. Liu S et al. (18) used CalliSpheres drug- eluting beads-bronchial artery chemoembolization embolization (DEB-BACE) to treat 21 refractory non-small cell lung cancer (NSCLC). After treatment the quality of life was significantly improved, and no serious adverse events such as spinal cord injury and cerebral embolism during the perioperative period. However, in the current case, the patient was significantly relieved only by simple embolization, which suggests that finding specific target vessels is of vital importance for killing tumors. Combined chemotherapy can achieve the effect of icing on the cake.

In addition, the patient received IMRT on day 2 following the intervention, and the tumor continued to regress during radiotherapy. After the 10^th^ radiotherapy session, the maximum diameter of the tumor was 20 mm. Theoretically, tumor cell hypoxia is exacerbated by embolization, which could promote the development of dormant carcinoma stem cells, sustain their potential for proliferation and differentiation, and reduce tumor sensitivity to radiotherapy. Further research is required to explore the process of continuous tumor regression in patients undergoing radiotherapy and how embolization alters the tumor microenvironment (19).

Although BAE is not a routine treatment per the National Comprehensive Cancer Network Guidelines for non-small cell lung cancer, its anticancer effect could be underestimated. Local interventional embolization combined with systemic therapy could be an excellent strategy to control the overall tumor load. The selection of suitable blood vessels is imperative. The timing of embolization and the mode of combination of constituents are topics for further research.

## Data availability statement

The original contributions presented in the study are included in the article/**Supplementary Material**. Further inquiries can be directed to the corresponding authors.

## Ethics statement

The studies involving human participants were reviewed and approved by the Ethics Committee of Shandong Cancer Hospital and Institute. The patient provided his written informed consent to participate in this study. Written informed consent was obtained from the individual(s) for the publication of any potentially identifiable images or data included in this article.

## Author contributions

XH and YM contributed to conception and design of the report. SZ wrote the first draft of the manuscript. JZ, XM, XH and YM wrote sections of the manuscript. All authors contributed to the article and approved the submitted version.
