# Supplementary figures and images for: Case Report: Bronchial artery embolization and chemoradiotherapy for central squamous cell lung carcinoma with rapid regression
Source: Front Oncol. 2022 Dec 14;12:1026087. doi: 10.3389/fonc.2022.1026087 (PMC9795177; doi:10.3389/fonc.2022.1026087)

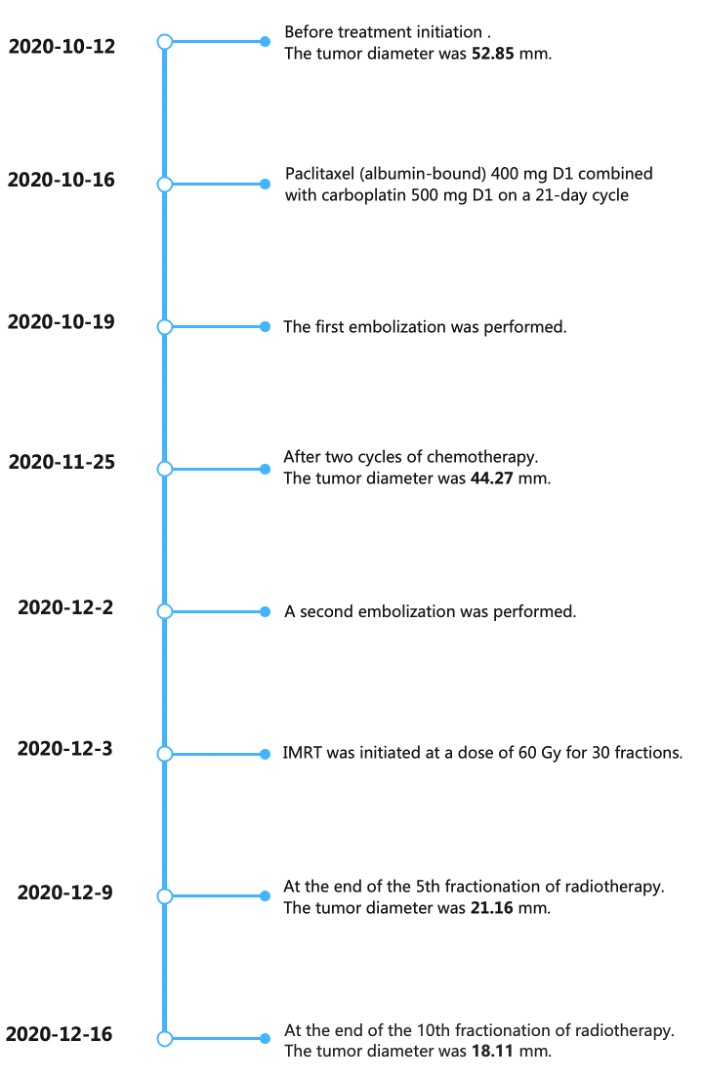

Supplement: Supplementary file 1 [file Image_1.jpeg]
